# Structural characterization of human importin alpha 7 in its cargo-free form at 2.5 Å resolution

**DOI:** 10.1038/s41598-021-03729-3

**Published:** 2022-01-10

**Authors:** S. Tsimbalyuk, C. M. Donnelly, J. K. Forwood

**Affiliations:** 1 School of Dentistry and Medical Sciences, Charles Sturt University, Wagga Wagga, NSW 2678 Australia; 2 School of Dentistry and Medical Sciences, Charles Sturt University, Room 2, National Life Sciences Hub, Wagga Wagga, NSW 2678 Australia

**Keywords:** X-ray crystallography, Protein transport

## Abstract

Shuttling of macromolecules between nucleus and cytoplasm is a tightly regulated process mediated through specific interactions between cargo and nuclear transport proteins. In the classical nuclear import pathway, importin alpha recognizes cargo exhibiting a nuclear localization signal, and this complex is transported through the nuclear pore complex by importin beta. Humans possess seven importin alpha isoforms that can be grouped into three subfamilies, with many cargoes displaying specificity towards these importin alpha isoforms. The cargo binding sites within importin alpha isoforms are highly conserved in sequence, suggesting that specificity potentially relies on structural differences. Structures of some importin alpha isoforms, both in cargo-bound and free states, have been previously solved. However, there are currently no known structures of cargo free importin alpha isoforms within subfamily 3 (importin alpha 5, 6, 7). Here, we present the first crystal structure of human importin alpha 7 lacking the IBB domain solved at 2.5 Å resolution. The structure reveals a typical importin alpha architecture comprised of ten armadillo repeats and is most structurally conserved with importin alpha 5. Very little difference in structure was observed between the cargo-bound and free states, implying that importin alpha 7 does not undergo conformational change when binding cargo. These structural insights provide a strong platform for further evaluation of structure–function relationships and understanding how isoform specificity within the importin alpha family plays a role in nuclear transport in health and disease.

## Introduction

The shuttling of macromolecules such as RNA and proteins between the cytoplasm and nucleus is an important and fundamental process for eukaryotic cells. The process is highly regulated, mediating a range of differentiation and developmental pathways, but is also targeted during viral infections and implicated in cancer pathogenesis^1–3^. Whilst molecules smaller than 40 kDa can diffuse passively through the nuclear pore complex (NPC), larger molecules require active transport^4^. The classical nuclear import pathway is mediated by specific interactions between proteins from the karyopherin family and cargo proteins harbouring a nuclear localization signal (NLS)^5^. Members of the IMPα are responsible for binding NLSs displayed within cargo, and through interaction with IMPβ, the heterotrimer is imported into the nucleus^6^. Upon entry, the cargo is released by RanGTP binding, and the importins are recycled back to the cytoplasm^7–9^.

IMPα proteins consist of three functional domains, an N-terminal IMPβ-binding (IBB) domain that mediates interaction with IMPβ, ten ARM domains that recognize and interact with cargo, and a C-terminal CAS domain (involving ARM 10) that mediates nuclear export and recycling^5,10,11^. The ten tandem ARM repeats are represented by three α-helices (H1, H2 and H3) spanning ~ 40 amino acids. Overall these ARM repeats form a bean-shaped molecule with the H3 helices defining the inside of the concaved surface. The inside groove harbour a number of Asn and Trp residues at the third and fourth turn of H3, and play roles in cargo binding^12^. The Trp and Asn are absent in ARM repeats 5 and 6, resulting in the segregation of IMPα binding regions into major (ARM 2–4) and minor (ARM 6–8) sites^12^. A monopartite NLS (consisting of a single basic region) can bind to both the major and minor sites of IMPα, whereas a bipartite NLS (consisting of two basic regions separated by a 10–12 amino acid linker) binds to both the major and minor sites^13^.

There are seven isoforms of human IMPα, grouped into three subfamilies that exhibit specificity for specific nuclear cargo^14^. The IMPα1 subfamily has the lowest sequence identity and conservation, and consists of IMPα1 and IMPα8 isoforms. IMPα1 isoform and mouse homologue IMPα2 have been extensively studied with multiple structures available describing classical monopartite binding^15^. The IMPα2 subfamily is comprised of two highly similar IMPα isoforms, IMPα3 and α4. While there are several structures of IMPα3 available, the structure of IMPα4 has not been solved to date^15^. Finally, the IMPα3 subfamily has the highest sequence homology and conservation among the subfamily members and includes IMPα5, α6, α7 isoforms^15^. Despite the high similarities, different tissue expression profiles have been observed, including the limited expression of IMPα6 exclusively in testis^16^. Furthermore, IMPα7 is critical for development in mice, with a knockout causing embryonic development to halt at the two-cell stage^17^. More recently, IMPα7 has been shown to play a critical role in regulating spermatogenesis and Sertoli cell function^18^.

Currently, there is only one structure of IMPα7 solved to date, and this is in its cargo bound state with the influenza PB2 protein (PDB 4UAD)^19^. As have been previously reported, no significant variation in the core ARM domains was observed upon binding of the cargo protein^20^. Here, we describe the first structure of IMPα7 NLS binding domain (ARMS 1–10) in the cargo-free state. We evaluate the similarities between IMPα isoforms from other subfamilies and compare the structures of cargo-free and bound structures.

## Materials and methods

### Protein constructs, protein expression and purification

The gene encoding IMPα7 ARM domains 1–10 (lacking the importin-beta binding (IBB) domain) (KPNA6, Uniprot ID O60684, residues 74-536) was codon optimized for *Escherichia coli* expression and cloned into the pET30a(+) vector at the BamHI site (Genescript, Picataway, NJ). The recombinant protein sequence incorporated the addition of the TEV protease amino acid sequence and a cleavable N-terminal His-tag. Plasmids were transformed in BL21(DE3) pLysS *E. coli* cells using the heat-shock method and were recombinantly expressed based on methods described previously for other importin isoforms^20^. The protein was purified using a Ni–NTA affinity column pre-equilibrated with His buffer A (50 mM phosphate buffer, 300 mM sodium chloride, 20 mM imidazole, pH 8.0) and eluted using a linear gradient of imidazole over five column volumes using His buffer B (50 mM phosphate buffer, 300 mM sodium chloride, 500 mM imidazole, pH 8.0). The protein was further purified using size exclusion chromatography on a Superdex 200 pg 26/600 column (GE Healthcare) using Tris-Buffered saline (50 mM Tris–HCl, 125 mM sodium chloride, pH 8.0). A single peak corresponding to a monomer was pooled together and analyzed on SDS-PAGE and concentrated using a 10 kDa MW centrifugal filter and stored at − 80 °C.

### Crystallization, data collection and processing

Crystallization trials were performed using 48 well crystallization plates with 1.5 μl protein mixed with 1.5 μl of reservoir solution, equilibrated over 300 μl reservoir solution using the hanging-drop vapour diffusion method. The IMPα7 protein crystallized at 15 mg/ml over a reservoir solution containing 0.1 M MES pH 6.5 and 12% PEG 20,000 at 18 °C. Rod-shaped crystals appearing in 30 days were cryoprotected in 25% glycerol and flash-cooled in liquid nitrogen. X-ray diffraction data from a single crystal was collected over 3600 images at 0.1^o^ oscillation at the Australian National Synchrotron MX2 beamline (Eiger X 16 M detector). The data were processed in iMosflm^21^, scaled in Aimless^22^ and phased using molecular replacement in Phaser^23^ with 4UAD^19^ as the search model. The structure was modelled and refined in Coot^24^ and Phenix^25,26^, respectively.

### PDB accession code

Coordinates and structure factors have been deposited in the PDB and released under accession code 7RHT.

## Results and discussion

### Structure of IMPα7 in cargo-free form

The cargo binding domain (ARMS 1–10) of IMPα7ΔIBB was successfully cloned and expressed, and crystals diffracting at Australia National Synchrotron MX2 beamline to 2.5 Å were indexed in the space group P21 21 21 with unit cell parameters of *a* = 64.85, *b* = 75.84, *c* = 88.97. The structure was solved using molecular replacement in Phaser^23^ using chain A of PDB model 4UAD^19^. One molecule of IMPα7 was present in the asymmetric unit, and following modelling and refinement in COOT^24^ and Phenix^25,26^, a model was produced with good stereochemistry and an R_work_ and R_free_ of 20.9% and 23.7% respectively. Full data collection and refinement statistics are presented in Table 1.

**Table 1:** Data collection and refinement statistics.

Data collection and processing	IMPα7 (PDB code: 7RHT)
Wavelength (Å)	0.9537
Resolution range (Å)	24.77–2.50 (2.60–2.50)
Space group	P 21 21 21
Unit cell (Å, ^o^)	64.85 75.84 88.97 90 90 90
Total reflections	77,300 (8824)
Unique reflections	15,096 (1703)
Multiplicity	5.1 (5.2)
Completeness (%)	97.2 (98.5)
Mean I/sigma(I)	8.1 (2.0)
Wilson B-factor Å^2^	38.58
R-merge	0.134 (0.958)
R-pim	0.09 (0.655)
**Refinement**	
Number of reflections	15,070
Number of R-free reflections	751
R-work %	20.9
R-free %	23.7
RMS(bonds) Å	0.003
RMS(angles), ^o^	0.50
**Ramachandran plot**	
Favoured (%)	97.87
Allowed (%)	2.13
Outliers (%)	0
**Validation**	
Clash score	1.66
PDB accession code	7RHT

**Figure 1:**
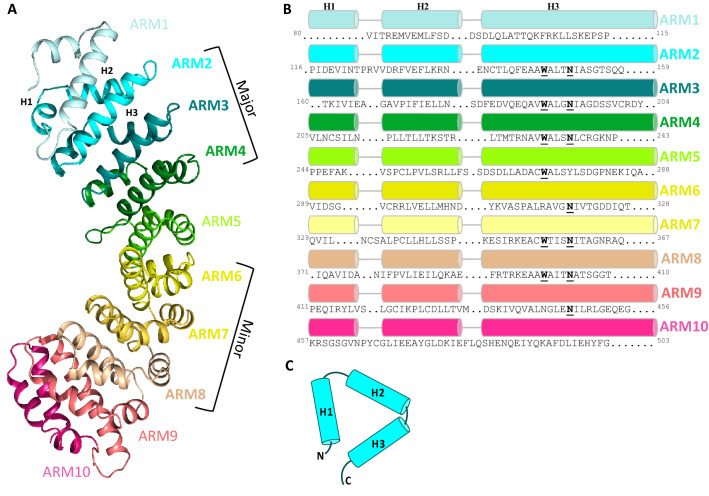
(**A**) Structure of IMPα7 ARM domains resolved to 2.5 Å resolution in cargo-free form shown in ribbon-cartoon format containing 10 ARM-repeats. (**B**) IMPα7 ARM domain structure-based sequence alignment with α-helices shown as colour-coded cylinders matching panel (**A**), with structural repeat H1, H2 and H3 indicated at the top. The residue numbers are shown in the beginning and at the end of each repeat. Presence of highly conserved Trp and Asn residues highlighted in bold and underline. (**C**) Each ARM repeat consists of three alpha helices: H1, H2 and H3, forming a stable triangular shape.

The final model of IMPα7ΔIBB consists of 424 residues (80–503) and 34 waters, with 79 helix-helix intramolecular interactions (analyzed in PDBSum)^27^. Overall, the structure exhibited a conserved topology and architecture similar to other IMPα isoforms. The structure is comprised of ten ARM domains (Fig. 1A,B), each consisting of three α-helices H1, H2 and H3 in a triangular arrangement (Fig. 1C), and overall forming a concave assembly^28,29^. Due to flexibility and lack of density at the N-terminus, the H1 of ARM1 could not be modelled. The inner concave surface of IMPα7 contained highly conserved Trp and Asn residues within H3 α-helices on ARMs 2–4 and 7–8, creating cargo NLS binding pockets at the major and minor sites, respectively^15^ (Fig. 1B).

### Minor cargo-induced structural changes observed in IMPα7

**Figure 2:**
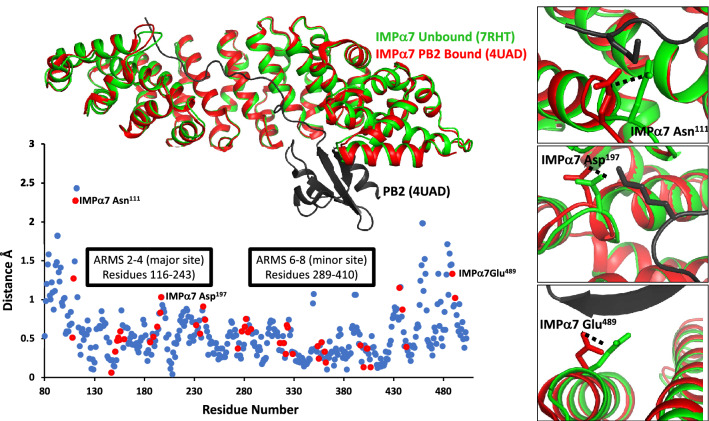
Structural alignment of IMPα7 in unbound (coloured green, PDB 7RHT) and bound (coloured red and PB2 NLS in black, PDB 4UAD^19^) forms. Graph inset represents distance differences between bound and unbound structures. Red dots reflect the positions within IMPα7 mediating PB2 binding. Minor changes in the positioning of some residues (listed in the graph inset) were observed in the cargo bound form, and these are presented in the right panels.

Studies have reported that importins may undergo conformational changes upon cargo binding, whilst other studies have reported little to no significant variation in the core ARM domains upon cargo binding^20^. For example, IMPβ undergoes structural changes upon binding to Ran and nuclear import cargo^8,30–32^. Similarly, IMPα1 has been shown to undergo conformational changes within the IBB domain to facilitate cargo binding^33^ and the flexibility within hinge region of IMPα3 has been reported to contribute to RCC1 specificity^34^. In contrast, structural comparisons between both unbound IMPα1 and IMPα3, and their requisite cargo bound forms with Henipavirus W proteins, revealed no major structural changes and similar positioning within the core ARM domains^20^. Since only one structure of IMPα7 in a cargo bound form has been solved to date (IMPα7 in complex with Influenza PB2 protein (PDB 4UAD)^19^), and here we describe the first structure of IMPα7 in an unbound form, we performed structural comparisons between these two structures to examine how these observations extend to IMPα7. Structural alignment using Superpose in CCP4^35^ revealed highly similar structures, with an r.m.s.d of 0.66 Å for IMPα7 mainchain residues of 80–503 (424 residues) (Fig. 2). We found that the positioning of ARM domains responsible for binding cargo at the major and minor sites (ARMS 2–4 and 6–8) were highly similar across both structures, whilst ARM domains at the extremities (ARMS 1, 9–10) exhibited minor structural changes that appeared to coincide with cargo binding (Fig. 2).

### IMPα7 structure comparisons with IMPα1, α3 and α5

**Figure 3:**
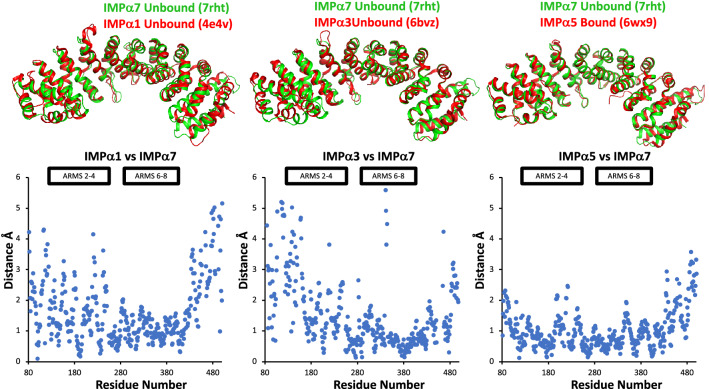
Structural comparisons of unbound IMPα7 with other IMPα subfamily members. IMPα1 (unbound, PDB 4e4v), IMPα3 (unbound, PDB 6bvz), IMPα5 (unbound structure remains to be determined, PDB 6wx9) isoforms were superimposed using CCP4 Superpose and the structural differences analyzed (see graph inset). The positions of the ARM domains 2–4 and 6–8, mediating cargo binding at the major and minor sites, respectively, are highlighted in bold.

Despite the relatively high sequence similarities and conserved residues at the cargo binding site, IMPα isoforms exhibit structural differences that potentially account for cargo specificity^15^. The structural basis for importin isoform specificity has been previously investigated in a limited number of studies^20,36^, and so we compared IMPα7 with IMPα isoforms from other subfamilies (Table 2). We found that IMPα7 was most structurally similar to IMPα5, ranging in r.m.s.d values of 1.3–1.5 Å (Table 2), whilst IMPα1 structures ranged from r.m.s.d values of 1.4–2.2 Å, and IMPα3 structures ranged from r.m.s.d values of 1.5–2.5 Å. We also found that comparing the unbound structure of IMPα1 with α7 revealed structural differences in the positioning at the major site (ARM 4) and ARMS 1, 9–10 (Fig. 3), whilst comparisons between unbound IMPα3 and α7 revealed structural differences at both the major and minor sites as well as ARM extremities. Finally, comparisons for IMPα5 (for which is there no unbound structure and therefore PDB 6wx9 was used) exhibited far fewer structural changes, localized mainly within the C-terminal ARM domains 9–10.

**Table 2:** Structural comparison of IMPα7 structure with other human IMPα isoforms.

PDB	z-score	r.m.s.d	Aligned Res	% Seq ID	IMPA	Subfamily
4uad	55.8	0.7	424	100	IMPα7	3
6wx9	53.7	1.3	423	86	IMPα5	3
4b18	53.6	1.4	424	86	IMPα5	3
2jdq	53.2	1.4	402	84	IMPα5	3
3tj3	52.6	1.5	424	86	IMPα5	3
7rg5	49.1	1.5	414	52	IMPα3	2
4uae	48.6	1.5	414	52	IMPα3	2
6bwb	48.6	1.5	414	52	IMPα3	2
6bwa	48.5	1.5	414	52	IMPα3	2
6bw9	48.4	1.5	414	52	IMPα3	2
6bvv	48.3	1.6	414	52	IMPα3	2
5xzx	47.5	1.6	413	52	IMPα3	2
6wx8	47.7	2.1	414	52	IMPα3	2
7jjl	47.9	2.2	411	52	IMPα3	2
6bvz	47.5	2.5	414	52	IMPα3	2
3fex	48.2	1.4	412	52	IMPα1	1
4wv6	49.4	1.5	415	52	IMPα1	1
3fey	49	1.5	415	52	IMPα1	1
7n8j	49	1.5	415	52	IMPα1	1
4e4v	46.7	2.2	415	52	IMPα1	1

**Figure 4:**
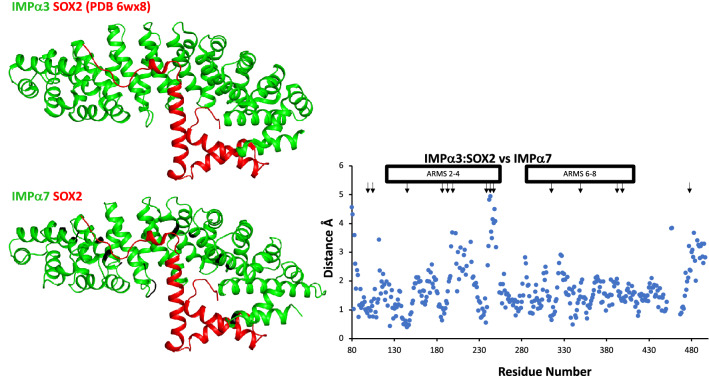
Structural alignment of unbound IMPα7 with IMPα3 in complex with SOX2 (PDB 6WX8). Phenix validation was used to analyze clash data. IMPα7 with merged SOX2 produced clashscore value of 20.7. The positions of clashing residues of IMPα7 are highlighted in black. Clashes are also presented in the graph, with clashes > 0.8 highlighted with black arrows.

Previous research has highlighted the importance of the positioning of ARM domains 7 and 8 for isoform specificity^20,36^. In addition, steric clashes between the cargo NLS and ARM7 and ARM8 domains of IMPα have been reported when aligning cargo-bound structures of IMPα2 and IMPα5 with IMPα3^36^. In this regard, to examine whether the IMPα7 structure described in this study could bind SOX2, we superimposed IMPα7 with IMPα3:SOX2 (PDB 6wx8) and examined the structures for possible steric clashes. We observed 62 atomic clashes (with a clash score of 0.8 or greater in Phenix validation) involving 14 residues with IMPα7 (Fig. 4). These clashes were observed both within the major and minor sites, and the ARM extremities (Fig. 4). These clashes would suggest that the binding between IMPα7 and SOX2 would be weaker than that observed between IMPα3 and SOX2, which is consistent with both a previous report showing a lack of detectable binding between IMPα7 and SOX2^36^ and the notion that differential positioning of ARM domains in the IMPα isoforms can confer specificity of cargo binding^20,36^. Moreover, the clashes observed at the major site within the superimposed model of IMPα7 and SOX2, together a previous report of IMPα5 binding with SOX2, is supported by the minor structural differences we observed between IMPα5 and IMPα7 at the major site (ARMS 2–4; Fig. 3).

## Conclusion

Here, we describe the first structure of human IMPα7 in cargo-free form. Structural analysis revealed that the IMPα7 protein exhibits the same structural architecture as other IMPα isoforms, and there were only minor conformational changes upon cargo binding. IMPα7 was most structurally similar to IMPα5, supporting their grouping within the same subfamily. The structural differences observed between IMPα3 and IMPα7 is consistent with previous studies highlighting the role of ARM domains 7 and 8 in mediating cargo specificity between these IMPα isoforms.
